# Late Recurrence of Spindle Cell Sarcoma in Association with *TPM3::NTRK1* Fusion

**DOI:** 10.1177/10668969251343223

**Published:** 2025-06-09

**Authors:** Xenia Parisi, Anindita Ghosh, Wei-Lien Wang, Nahir Cortes-Santiago, L. Jeffrey Medeiros

**Affiliations:** 1 Department of Hematopathology, 461915 The University of Texas MD Anderson Cancer Center Division of Pathology and Laboratory Medicine, Houston, TX, USA; 2 Department of Anatomical Pathology, 461915 The University of Texas MD Anderson Cancer Center Division of Pathology and Laboratory Medicine, Houston, TX, USA; 3 Department of Pathology and Immunology, Texas Children's Hospital, Houston, TX, USA

**Keywords:** *NTRK*, receptor tyrosine kinase pathway, spindle cell, sarcoma, larotrectinib

## Abstract

We report a 31-year-old woman with a history of low-grade, undifferentiated spindle cell sarcoma of the hand diagnosed at 10 years of age and subsequently amputated. The patient remained under surveillance without recurrence for over 20 years before she presented with multiple new masses in the skin of the head and neck. Biopsies confirmed recurrent sarcoma. Imaging studies revealed metastases within the lungs and peri-pancreatic tissue. Molecular analysis of these lesions showed a *TPM3::NTRK1* fusion, and the patient started treatment on larotrectinib, on which she now remains, relapse and recurrence-free at 40 months. In presenting this patient, we hope to draw attention to the potentially overlooked provisional category of *NTRK*-rearranged spindle cell neoplasms that has been added to the fifth edition of the World Health Organization (WHO) classifications of pediatric tumors and soft tissue and bone tumors. While these neoplasms constitute only an estimated 1% of pediatric soft tissue neoplasms, discovering *NTRK* abnormalities in these lesions opens an important line of targeted therapy options, i.e., NTRK inhibitors. We hope to also show the community the potential for *NTRK*-rearranged spindle cell neoplasms to present with late local recurrence and metastases.

## Introduction

The classification of fibroblastic and myofibroblastic pediatric tumors has expanded tremendously with our understanding of their molecular biology.^1,2^ Concurrently, target-based drug design efforts have developed elegant, tissue-agnostic agents that have transformed the management of previously “orphan” cancers into druggable ones. As a result, including provisional categories such as *NTRK*-rearranged spindle cell neoplasms becomes essential for triaging patients to the safest and most effective management options. Here we report a patient with sarcoma positive for a *TPM3::NTRK1* fusion that developed recurrence and metastases 22 years after initial diagnosis and amputation, but which has responded well to therapy with larotrectinib.

## Clinical Presentation

A 31-year-old woman with a remote history of a low-grade, undifferentiated spindle cell sarcoma of the hand, diagnosed and treated by amputation at 10 years of age, presented with multiple palpable masses growing on the scalp, base of the neck and along the midback. She underwent an excisional biopsy and was diagnosed with multifocal desmoid tumors. Computed tomographic imaging demonstrated additional para-mediastinal and pulmonary lesions along with a 3.6 cm mass involving peri-pancreatic soft tissue and questionably involving the pancreatic head, for which her care was transferred to a tertiary cancer center.

**Figure 1.**
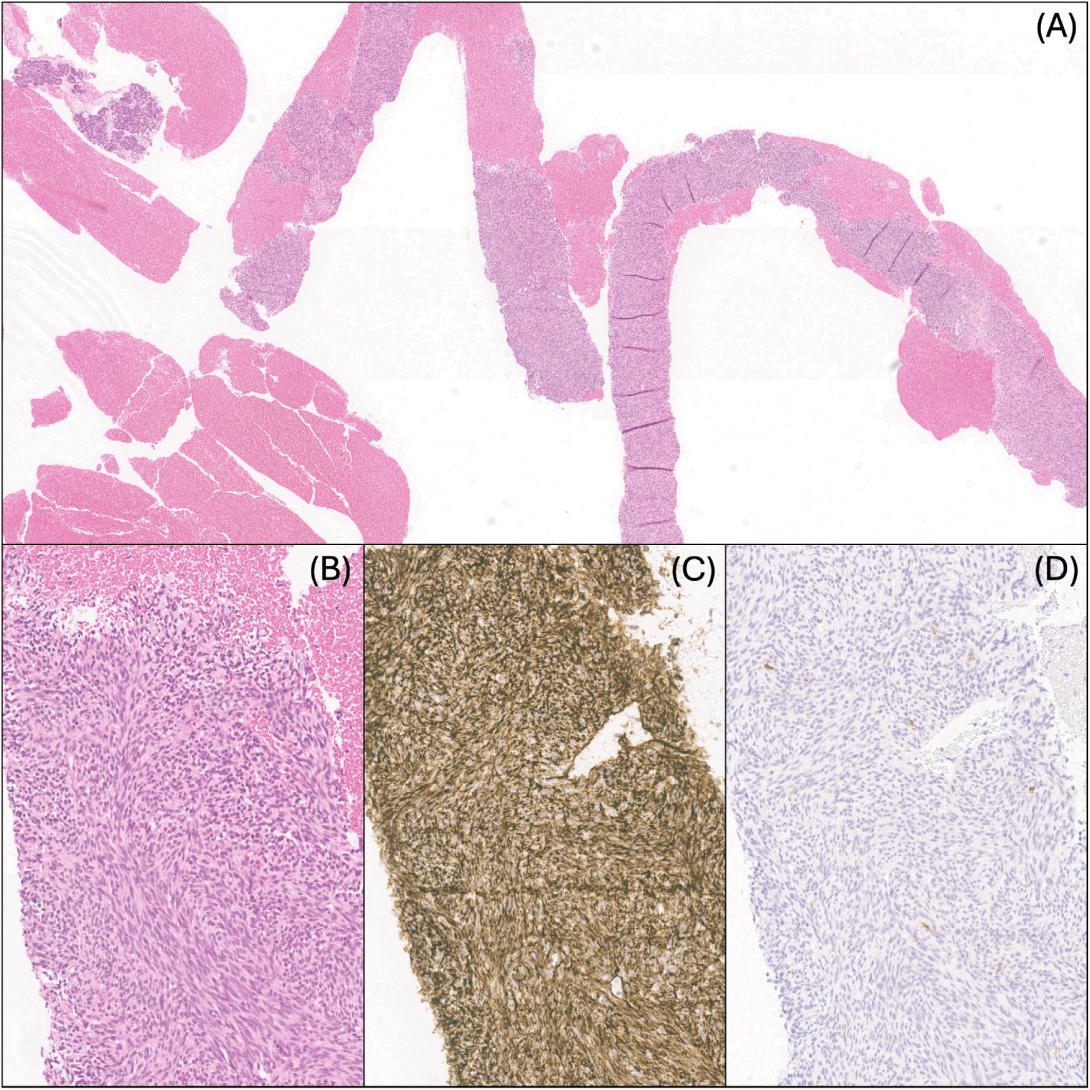
Core needle biopsy fragments of the peri-pancreatic lesion: (A) H&E, 40×, (B) H&E, 200×, (C) CD34, ×200, and (D) S100, ×200.

A biopsy of the peri-pancreatic mass showed core needle-shaped fragments of a monomorphic spindle cell neoplasm arranged in a solid, fascicular, and vague storiform pattern (Figure 1). The neoplasm had a low mitotic index (<1 mitotic figure per high-power field). There was no evidence of necrosis. Hemangiopericytoma-like blood vessels and perivascular hyalinization were not prominent. Immunohistochemical analysis was performed using fixed, paraffin-embedded tissue sections. The neoplastic cells were positive for CD34 (diffuse), CD10 (MME), and vimentin, and were negative for pan-keratin, EMA, CD56, S100 protein, desmin, myogenin, MyoD1, BCOR, TLE1, STAT6, SMA, HMB45, SOX10, HHV8, DOG1, and p53. H3k27me3 was retained. Ki-67 showed a proliferation index of approximately 15%. A review of the patient's initial tumor specimen and medical records, consisting of an H&E section and reported immunohistochemical findings (positive for vimentin; negative for SMA, desmin, and S100 protein), confirmed that the neoplasm in the peri-pancreatic soft tissue was consistent with the hand tumor resected in childhood. The recurrent neoplasm was assessed by fluorescence in situ hybridization analysis; there was no evidence of *PDGFRB* or other sarcoma-associated rearrangements. Classical cytogenetic analysis was not performed but next-generation sequencing analysis identified a *TPM3::NTRK1* fusion between *TPM3* exon 8 (NM_152263) and *NTRK1* exon 10 (NM_002529). Additional molecular studies showed *CDKN2A/B* loss, *KMT2C(MLL3)* Q4514fs*108, and *NUP98* exon 4 truncation. The neoplasm was microsatellite (MSI) stable with an intermediate tumor burden (6 mutations/megabase). The patient underwent surgical excision of all discrete lesions and was started on larotrectinib therapy. She continues treatment and remains free of recurrences or evidence of progressive disease at 40 months.

## Discussion

Undifferentiated spindle cell neoplasms with *NTRK*-rearrangement (or activating mutations in *RAF1*, *BRAF*, *RET, MET,* and others) are rare, recently described lesions in children. These mesenchymal tumors typically present as a painless, slow-growing mass on the extremities, but can also occur on the trunk or head and neck region.^3,4^ Histologically, *NTRK*-rearranged (*NTRK*-R) spindle cell neoplasms most commonly are composed of a monomorphic, haphazardly arranged spindle cell population accompanied by distinctive stromal and perivascular hyalinization and hemangiopericytoma-like vasculature; however, in many instances, the morphologic findings overlap with other entities that are described.^5,6^ Despite their malignant nature, *NTRK*-R spindle cell neoplasms generally have favorable long-term outcomes.^3,4,6^ Although rare, these neoplasms can recur many years after initial treatment, and long-term follow-up of affected patients is required.

The patient we report is notable for the long latency interval between initial diagnosis and treatment, and recurrence, nearly 20 years. The recurrence of this patient's low-grade sarcoma manifested as multiple masses in the scalp, along the back, the lungs and peri-pancreatic soft tissue. The diagnosis of any sarcoma can be challenging due to nuanced histologic findings that often overlap in a spectrum between multiple named entities. The differential diagnosis for desmoid tumor, as this patient was originally designated, potentially ought to include infantile fibrosarcoma, congenital fibromatosis, rhabdomyosarcoma, synovial sarcoma, malignant peripheral nerve sheath tumor, solitary fibrous tumor, and neurofibroma. In these diagnoses, or when a definitive diagnosis cannot be established, evaluating for *NTRK* fusions may be considered, as their presence could support the use of targeted tyrosine kinase inhibitors such as larotrectinib, entrectinib, and repotrectinib. However, given the rarity of *NTRK* alterations in sarcomas, the clinical utility of testing remains limited to patients where either front-line therapy has failed, cannot be tolerated, or for patients who otherwise may require a disfiguring surgery, or with metastatic disease as the patient reported here.^7–9^

Xu et al^10^ reported two other published incidences of relapsed/metastatic disease in patients with *NTRK*-R. One instance was a newborn girl diagnosed with a 9 cm congenital infantile fibrosarcoma of the subcutis and dermis over the face and neck. The lesion was described as having had a mitotic index of 30 per 10 high-power fields, nuclear atypia and necrosis and carried an *ETV6::NTRK3* fusion. On examination of the resection specimen, there was a microscopically positive margin, and she was found later to have local recurrence and distant metastasis to the brain. Despite multiple cycles of vincristine/actinomycin-D/cyclophosphamide; ifosfamide/doxorubicin, and ifosfamide/etoposide, her disease progressed. She was then started on larotrectinib (first-generation NTRK inhibitor). The patient had an incomplete response, and molecular analysis in a biopsy of the recurrent lesion showed a new *NTRK3* G623R mutation. Drug resistance was attributed to this mutation given previous demonstrations by others that the *NTRK3* G623R mutation results in a steric inhibition between the arginine side chain of mutant TRKC and the hydroxypyrrolidine group of larotrectinib.^11–12^ The other patient in their report was a 4-year-old boy who had a 4 cm lipofibromatosis-like neural tumor of the mandibular bone and soft tissue. This tumor was reported to have a mitotic index of 4 per 10 high-power fields without nuclear atypia or necrosis and an *LMNA::NTRK1* fusion. He experienced local recurrence 10.5 years after the initial resection. No repeat molecular information was made available for this patient. Ultimately, additional studies on the nature of drug resistance and variants of targeted tyrosine kinase inhibitors are needed but NTRK inhibitors provide a useful treatment option.

## Conclusions

This report highlights the potential for late recurrence of *NTRK*-R spindle cell neoplasms, even decades after initial treatment, and underscores the importance of ongoing surveillance in patients with a history of soft tissue sarcomas. The emergence of targeted therapies, such as NTRK inhibitors, offers hope for improved outcomes in patients with metastatic disease, particularly those patients with neoplasms that carry molecular targets amenable to tailored treatment.
